# Subjective Cognitive Decline May Be Associated With Post-operative Delirium in Patients Undergoing Total Hip Replacement: The PNDABLE Study

**DOI:** 10.3389/fnagi.2021.680672

**Published:** 2021-06-11

**Authors:** Xu Lin, Fanghao Liu, Bin Wang, Rui Dong, Lixin Sun, Mingshan Wang, Yanlin Bi

**Affiliations:** ^1^Department of Anesthesiology, Qingdao Municipal Hospital Affiliated to Qingdao University, Qingdao, China; ^2^Department of Anesthesiology, Nanjing Drum Tower Hospital, Nanjing, China

**Keywords:** subjective cognitive decline, post-operative delirium, total hip replacement, cerebrospinal fluid, biomarker

## Abstract

**Objective:** Subjective cognitive decline (SCD) is associated with an increased risk of clinical cognitive disorders. Post-operative delirium (POD) is a common complication after total hip replacement. We aimed to investigate the relationship between SCD and POD in patients undergoing total hip replacement.

**Methods:** Our study recruited 214 cognitively intact individuals from the Perioperative Neurocognitive Disorder And Biomarker Lifestyle (PNDABLE) study in the final analysis. SCD was diagnosed with Subjective Cognitive Decline Scale (SCDS), Mini-Mental State Examination (MMSE), and Montreal Cognitive Assessment (MoCA). The incidence of POD was evaluated by using Confusion Assessment Method (CAM), and POD severity was measured by using the Memorial Delirium Assessment Scale (MDAS). Preoperative cerebrospinal fluid (CSF) Aβ40, Aβ42, T-tau, and P-tau levels were measured by enzyme-linked immune-sorbent assay (ELISA).

**Results:** Overall, the incidence of POD was 26.64% (57/214), including 32.43% (36/111) in the SCD group and 20.39% (21/103) in the NC group. With the increase of age, the incidence of POD in all age groups increased (*P* < 0.05). Logistic regression analysis showed that after adjusting for SCD, Aβ42, Aβ40, P-tau, and T-tau, SCD (OR 2.32, CI 1.18–4.55, *P* = 0.01) and the increased CSF level of P-tau (OR 1.04, CI 1.01–1.06, *P* < 0.001) were risk factors for POD, while the level of aβ42 (OR 0.99, CI 0.99–1.00, *P* < 0.001) was a protective factor for POD.

**Conclusion:** SCD is one of the preoperative risk factors for POD.

**Clinical Trial Registration:** This study was registered at China Clinical Trial Registry (Chictr200033439).

## Introduction

Post-operative delirium (POD) is a common complication in the surgical patients. It is characterized by acute changes in the patients' mental state, involving impairment of cognition, attention, and consciousness levels, and tends to occur within 1 week after surgery (or before discharge) (Evered et al., 2018). POD is affected by a variety of factors, with the incidence ranging from 4 to 65% (Rudolph and Marcantonio, 2011), and 17% after total hip replacement (Oh et al., 2021). Previous studies have confirmed that POD has a high morbidity and mortality and at the same time reduces the quality of patients' life, prolongs the length of hospital stay, and increases the burden on families and society (Eckenhoff et al., 2020). So far, the pathogenesis of POD is still unclear. It was found that biomarkers such as amyloid (Aβ) and Tau protein in cerebrospinal fluid (CSF) have become strong predictors of POD (Jia et al., 2019; Bassil et al., 2020). However, there was no independent preoperative subjective cognitive status to predict POD. Therefore, it is crucial to identify an independent preoperative subjective cognitive status associated with POD that may be predicted in the preoperative settings.

Subjective cognitive decline (SCD) refers to a condition in which an individual's memory and/or other cognitive abilities are significantly reduced relative to one's previous performance level in the absence of an objective neuropsychological deficit (Jessen et al., 2014). The evidence shows that SCD increases the risk of future pathological cognitive declining (Cheng et al., 2017). However, to date, preoperative SCD has not been assessed as a risk factor of POD. Moreover, there is hardly any research on the relationship between SCD and POD and their related mechanisms.

Therefore, we planned to conduct a prospective, observational cohort study to investigate the relationship between SCD and POD and their related mechanisms, as well as whether there was a difference in the incidence of POD in SCD patients aged from 40 to 90 years old, so as to find a new way for the early prevention of POD. For these purposes, we make the hypothesis that preoperative SCD is a risk factor of POD in patients and that the significance of SCD may vary in different age groups. Three analyses were then performed. Firstly, the relationship between SCD and POD was assessed. Secondly, the relationship between Aβ40, Aβ42, T-tau, P-tau, and POD or SCD was analyzed. Thirdly, the incidence of SCD and POD in patients of different age groups was observed and analyzed.

## Materials and Methods

### The PNDABLE Study

Participants were recruited from the Perioperative Neurocognitive Disorder And Biomarker Lifestyle (PNDABLE) study, which is a large cohort study conducted in 2018 to analyze the risk factors and biomarkers of perioperative neurocognitive impairment in the Han population in northern China for the early diagnosis and prevention of the disease. The study has been registered with the China Clinical Trial Registry (clinical registration number: Chictr200033439), and ethical approval for this study (Ethical Committee N°2020 PRO FORMA Y number 005) was provided by the Ethical Committee Qingdao Municipal Hospital affiliated to Qingdao University, Qingdao, China (Chairman Prof Yang), on May 21, 2020. All patients were informed of the purpose of participation in the study and of the procedure (blood and CSF collection) and had signed an informed consent form prior to inclusion. Cognitive function was evaluated by subjective cognitive decline scale (SCDS), Mini-Mental State Examination (MMSE), and Montreal Cognitive Assessment (MOCA); the patients were diagnosed as normal cognitive (NC), SCD, mild cognitive impairment (MCI) and Alzheimer's disease (AD) according to the different scores. The patients of the NC group and SCD group were selected as the main study objects.

### Study Participants

This is a prospective, observational, cohort study of patients undergoing total hip replacement. Three hundred eligible patients who were between 40 and 90 years of age and scheduled to have total hip replacement under combined spinal and epidural anesthesia, between June 2020 and December 2020 in Qingdao Municipal Hospital affiliated to Qingdao University, were included in this study. The inclusion criteria of this study include (1) age 40–90 years; (2) Han Nationality Patients in north China; (3) American Society of Anesthesiologists (ASA) score 1 or 2; (4) preoperative cognitive status was good with no language communication disorder; and (5) educational level was enough to complete preoperative cognitive function test. The exclusion criteria include (1) central nervous system infection, head trauma, epilepsy, multiple sclerosis, and other major neurological diseases; (2) major psychological dysfunction; (3) severe systemic diseases (such as malignant tumors) that may affect the levels of POD biomarkers (Aβ and Tau) in CSF; (4) genetic family history; (5) preoperative MMSE scores of 23 or less or MOCA scores of 26 or less; (6) ASA score [a global score that assesses the physical status of patients before surgery, ranging from 1 (normal health) to 5 (moribund)] (Davenport et al., 2006) >2; (7) severe visual and hearing disorders; and (8) unwillingness to comply with the protocol or procedures.

**Figure 1:**
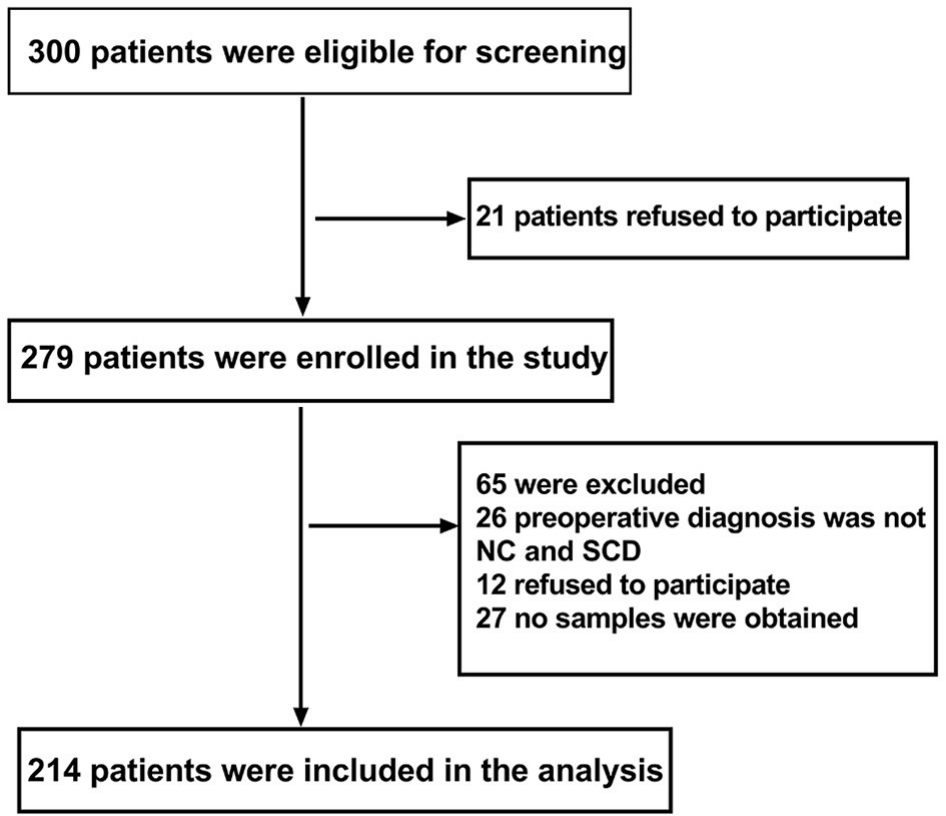
Flow diagram. The flow diagram shows that 300 patients were initially screened for the studies, and 214 patients were finally included in the data analysis.

Data of 214 patients were analyzed in this study (see Figure 1, flow diagram).

### Neuropsychological Testing

SCDS, MMSE, and MOCA scores were used to evaluate the cognitive function of the patients the day before the operation by a neurologist. Patients were asked whether their memory and/or other cognitive abilities were significantly reduced relative to their previous performance level in the absence of an objective neuropsychological deficit (Jessen et al., 2014), which was for diagnosing SCD, meanwhile, by the SCDS: (1) Compared with the past, the ability of conscious memory decreased which worried patients, which was the basic condition for diagnosis of SCD; (2) other conditions that ultimately determine SCD include normal MMSE scores of 24 or more, MoCA scores of 27 or more, and exclusion from other neuropsychiatric disorders, medical disorders, and drug or other substance abuse (Jorm et al., 1997; Jessen et al., 2014; Molinuevo et al., 2017). POD was defined by the Confusion Assessment Method (CAM), and POD severity was measured using the Memorial Delirium Assessment Scale (MDAS) (Inouye et al., 1990; Schuurmans et al., 2003) at 10 a.m. and 2 p.m. twice a day on 1–7 days (or before discharge) by an anesthesiologist post-operatively. The above assessments performed by a neurologist and an anesthesiologist were not involved in intraoperative management of the patients. The diagnosis of POD included the following four clinical criteria: (1) acute onset and fluctuation process; (2) inattention; (3) disorganized thinking; and (4) change of consciousness level. POD can be diagnosed if it meets the standards 1, 2, and 3 or 4 at the same time. The CAM and MDAS in Chinese research have been proven to have good reliability and validity (Leung et al., 2008; Shi et al., 2014). Therefore, CAM and MDAS positive scores on patients post-operatively on 1–7 days (or before discharge) were recorded.

### Anesthesia and Surgery

All patients who underwent total hip replacement under combined spinal and epidural anesthesia used the same surgical team to avoid the impact of different surgical techniques. During anesthesia, oxygen saturation, electrocardiography, non-invasive blood pressure, and pulse oximetry were continuously monitored, which were recorded at fixed intervals of 3 min. However, glucocorticoid drugs, dexmedetomidine non-steroidal analgesics, and midazolam were avoided during surgeries.

Post-operatively, the visual analog scale (VAS) score of 0–10 (lower score indicating lower level of pain) (Chung et al., 2016) was used to assess the pain at the same time. Patient-controlled intravenous analgesia (PCIA) was used post-operatively for 48 h by all patients. The PCIA opioid consisted of 2.5 μg·kg^−1^ sufentanil and 5 mg tropisetron (total volume of 100 ml, including 0.9% normal saline, bolus 2 ml, basal rate 2 ml/h, and lockout time 15 min). If patients need it, they were given non-opioid drugs for analgesia, which was recorded.

### Sample Collection

The CSF (2 ml) was collected in a polypropylene centrifugal tube during spinal-epidural joint block prior to administration of the local anesthetic, which were centrifuged immediately at 2000 g for 10 min at room temperature (Bakr et al., 2017; Pérez-Ruiz et al., 2018) and then stored at −80°C for further analysis.

### Elisa

The concentrations of Aβ40, Aβ42, T-tau, and P-tau were detected from 1.5 ml CSF using Aβ40 (BioVendor, Ghent, Belgium Lot: No. 292-6230), Aβ42 (BioVendor, Ghent, Belgium Lot: No. 296-64401), P-tau (BioVendor, Ghent, Belgium Lot: QY-PF9092), and T-tau (BioVendor, Ghent, Belgium Lot: No. EK-H12242) assay kit in accordance with the manufacturer's protocol. Finally, the optical density value (OD value) of each hole was measured at the wavelength of 450 m with an enzyme marker (EnSpire, PerkinElmer, Waltham, MA, USA) (Bakr et al., 2017; Pérez-Ruiz et al., 2018). All samples were assayed by the same laboratory assistant who was blinded to the group assignment.

### Study Size

No formal power analysis was performed, but we have a large cohort size which has included 1,905 patients in the PNDABLE study to decrease the risk of underpowered analyses.

### Data Analysis

SPSS statistical software, version 21.0 (SPSS, Inc., Chicago, IL, USA), and GraphPad Prism software, version 6.01 (GraphPad Software, Inc., La Jolla, CA, USA), were used for data analysis. The K–S test was used to determine whether the measurement data conformed to the normal distribution. The measurement data that conformed to the normal distribution was expressed by mean ± standard deviation (SD); the median and interquartile range (IQR, 25–75 percentile) or a number (%) to express the data. Independent sample *t*-test was used for comparison among groups, and χ^2^ test was used for counting data. Binary logistic regression analysis of SCD and POD, Aβ40, Aβ42, T-tau, and P-tau was performed. SCD, Aβ40, Aβ42, T-tau, and P-tau based on the univariate analysis were chosen as covariates in multivariate logistic regression analysis. *P* < 0.05 was statistically significant.

## Results

### Characteristics of Included Participants in PNDABLE

This study enrolled 300 participants. Two hundred fourteen (*n* = 214) were eligible for analysis, and 86 participants were excluded. The criteria for exclusion are shown in Figure 1. The demographic and clinical data are summarized in Table 1.

**Table 1:** Characteristics of participants.

	**SCD group (*N* = 111)**	**NC group (*N* = 103)**	***P*-value**
Age (year), mean ± SD	60.52 ± 10.63	63.06 ± 10.50	0.08
Male, n (%)	57 (51.35)	62 (60.19)	0.19
Years of education, n (%)			
0	8 (7.21)	7 (6.80)	0.10
1–9	49 (44.14)	45 (43.69)	0.57
10–13	26 (23.42)	25 (24.27)	0.35
14–17	18 (16.22)	17 (16.50)	0.21
>17	10 (9.01)	9 (8.74)	0.17
Height (cm), mean ± SD	169.61 ± 7.23	168.41 ± 5.43	0.86
Body weight (kg), mean ± SD	68.23 ± 8.63	65.17 ± 10.72	0.72
BMI (kg/m^2^), mean ± SD	26.23 ± 3.61	24.62 ± 2.53	0.08
ASA class, n (%)			
I	48 (43.24)	45 (43.69)	0.77
II	63 (56.76)	58 (56.31)	0.55
Dependence on smoking (n), n (%)	31 (27.93)	35 (33.98)	0.34
Alcohol abuse, n (%)	31 (27.93)	33 (32.04)	0.51
Coronary heart disease, n (%)	33 (29.73)	35 (33.98)	0.51
Hypertension, n (%)	38 (34.23)	40 (38.83)	0.49
Diabetes, n (%)	15 (13.51)	18 (17.48)	0.42
Family history of dementia, n (%)	5 (4.50)	4 (3.88)	1.00
Time of anesthesia (min), mean ± SD	141.38 ± 25.41	147.82 ± 23.41	0.76
Time of surgery (min), mean ± SD	133.14 ± 25.35	136.76 ± 31.73	0.88
Estimated volume of infusion (ml), mean ± SD	911.27 ± 33.86	922.45 ± 36.89	0.74
Estimated blood loss (ml), mean ± SD	111.97 ± 5.45	120.28 ± 7.79	0.59
Preoperative the highest MMSE score, median, and 25–75 percentile	24 (22–27)	25 (23–28)	0.31
Preoperative the highest MoCA score, median, and 25–75 percentile	27 (25–29)	28 (26–30)	0.18
Post-operative the highest MDAS score, median, and 25–75 percentile	13 (10–15)	12 (9–15)	0.79
Post-operative the highest VAS score, median, and 25–75 percentile	2 (1–3)	3 (2–5)	0.61

*The length of anesthesia was defined from the time that the anesthesiologists started the spinal anesthesia in the patients to the time when the patients were sent to the post-anesthesia care unit. The length of surgery was defined from the time of initial incision to the time of the closure of the skin. BMI, Body Mass Index; ASA, American Society of Anesthesiologists; cm, centimeter; min, minute; kg, kilogram; ml, milliliter; SD, standard deviation; MMSE, Mini-Mental State Examination; MOCA, Montreal cognitive assessment; MDAS, Memorial Delirium Assessment Scale; VAS, visual analog scale*.

The incidence of POD was observed in 26.64% (*n* = 57/214), with 32.43% (*n* = 36/111) in the SCD group and 20.39% (*n* = 21/103) in the NC group. Among the patients diagnosed with SCD, there was no significant difference in preoperative MMSE score [24 (22–27)] compared with patients diagnosed with NC [25 (23–28), *P* = 0.31]. Similarly, the preoperative MoCA score [27 (25–29)] in the SCD group was not significantly different from the score [28 (26–30), *P* = 0.18] in the NC group. In this study, it has been found that the POD and its severity were primarily diagnosed by CAM and MDAS scores on post-operative day 1 and day 2, which is consistent with previous studies (Lin et al., 2020). Moreover, MDAS scores [13 (10–15)] were not significantly different from the patients who were diagnosed as NC [12 (9–15), *P* = 0.79]. Additionally, the post-operative highest VAS scores are the same in the SCD group 2 (1–3) and NC group [3 (2–5), *P* = 0.61].

### Comparison of CSF Biomarker Levels Between Two Groups

Compared with the NC group, the differences in CSF levels of Aβ40, Aβ42, T-tau, and P-tau of the SCD group were statistically significant (*P* < 0.05), as shown in Table 2.

**Table 2:** Comparison of CSF biomarker levels between the two groups.

**Biomarkers**	**SCD group (*N* = 111)**	**NC group (*N* = 103)**	***P*-value**
Aβ40 (pg/ml, ± s)	4751.09 ± 2425.44	6233.88 ± 3450.97	<0.001
Aβ42 (pg/ml, ± s)	186.81 ± 136.24	233.33 ± 123.59	0.009
P-tau (pg/ml, ± s)	41.42 ± 16.99	35.43 ± 12.23	0.003
T-tau (pg/ml, ± s)	224.55 ± 146.39	167.56 ± 71.13	<0.001

*SCD, subjective cognitive decline; NC, normal cognitive; CSF, cerebrospinal fluid*.

### Logistic Regression Analysis of the Influencing Factors of POD and SCD

In this study, logistic regression analysis showed that patients with SCD were more prone to POD. SCD and the increased CSF level of P-tau were risk factors for POD; however, the increased CSF level of Aβ42 was a protective factor for POD by univariate analysis. After adjustment for SCD, Aβ42, Aβ40, P-tau, and T-tau, multivariate logistic regression analysis showed that SCD and the increased CSF level of P-tau were still risk factors for POD; the increased CSF level of Aβ42 was still a protective factor for POD, as shown in Table 3.

**Table 3:** Analyze the influencing factors of POD and SCD by logistic regression.

**Factors of interest**	**Unadjusted**		**Adjusted**	
	**OR (95%CI)**	***P*-value**	**OR (95%CI)**	***P*-value**
SCD	1.87 (1.01–3.49)	0.04	2.32 (1.18–4.55)	0.01
Aβ42	0.10 (0.99–1.00)	0.00	0.99 (0.99–1.00)	0.00
Aβ40	1.00 (0.99–1.00)	0.07	1.00 (0.99–1.00)	0.04
P-tau	1.04 (1.02–1.06)	<0.001	1.04 (1.01–1.06)	0.00
T-tau	1.00 (1.00–1.01)	0.00	1.00 (1.00–1.01)	0.01

*OR, relative risk; CI, confidence interval. After adding SCD, Aβ42, Aβ40, P-tau, T-tau, multivariate regression analysis was performed*.

### Relationship Between SCD and Biomarkers in CSF by Logistic Regression

The SCD was taken as the dependent variable; logistic regression analysis was carried out to explore the influencing factors. The results showed that the CSF levels of P-tau and T-tau were risk factors for SCD by univariate analysis. After adjustment for Aβ42, Aβ40, P-tau, and T-tau, the CSF levels of P-tau and T-tau were still risk factors for SCD by multivariate logistic regression analysis, as shown in Table 4.

**Table 4:** Relationship between SCD and biomarkers in CSF by logistic regression.

**Biomarkers**	**Unadjusted**		**Adjusted**	
	**OR (95%CI)**	***P*-value**	**OR (95%CI)**	***P*-value**
Aβ42	1.00 (0.99–1.00)	0.01	1.00 (0.99–1.00)	0.02
Aβ40	1.00 (0.99–1.00)	0.00	1.00 (0.99–1.00)	0.00
P-tau	1.03 (1.01–1.05)	0.01	1.04 (1.01–1.06)	0.00
T-tau	1.01 (1.00–1.01)	0.00	1.01 (1.00–1.01)	<0.001

*OR, relative risk; CI, confidence interval; CSF, cerebrospinal fluid. After adding Aβ42, Aβ40, P-tau, T-tau, multivariate regression analysis was performed*.

### Comparison of POD Incidence in Different Age Groups With SCD

**Figure 2:**
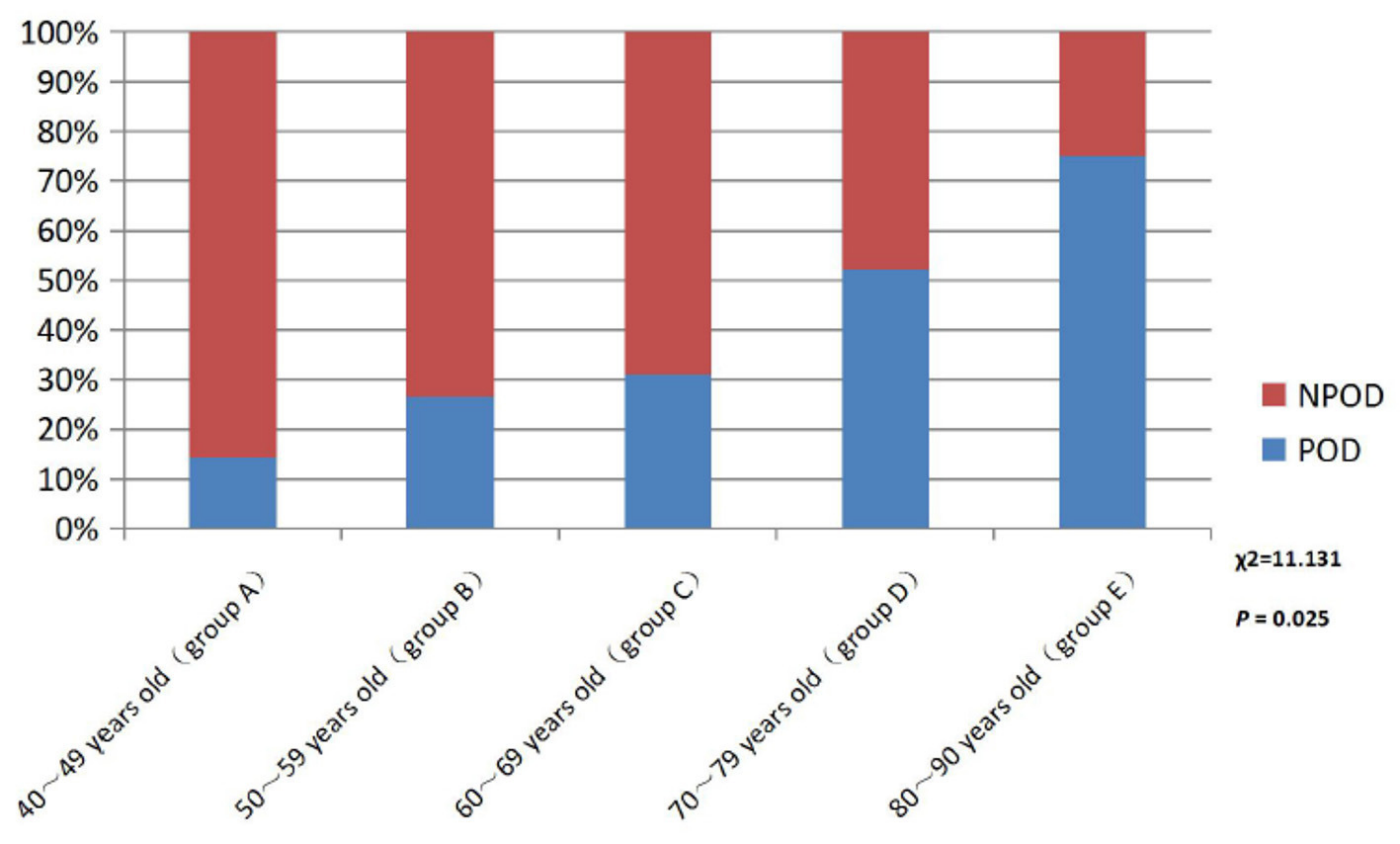
Comparison of POD incidence in different age groups patients with SCD. POD, post-operative delirium; NPOD, non-post-operative delirium.

There were no statistical significance differences in the mean age between the SCD and NC group in Table 1. Further, the patients with SCD were divided into five different age groups: 40–49 years old (Group A), 50–59 years old (Group B), 60–69 years old (Group C), 70–79 years old (Group D), and 80–90 years old (Group E). The results showed that the incidence of POD increased with age in SCD patients, as shown in Figure 2.

## Discussion

In this study, we found that SCD is one of the preoperative risk factors for POD.

POD is a syndrome with subjective variability and numerous influencing factors, and it is a product of the interaction of patients' demographic factors, basic disease factors, and anesthesia and surgery factors (Aldecoa et al., 2017). Its pathogenesis is complex, including the cholinergic theory of the cholinergic nerve, the theory of inflammatory response, the theory of exogenous toxin and free radical damage, the theory of Tau protein hyperphosphorylation, and the theory of Aβ protein abnormal deposition.

Previous studies have confirmed that the concentration of biomarkers such as Aβ42, Aβ40, T-tau, and P-tau in patients can be used as an important basis for clinical research and diagnosis of POD (McKhann et al., 2011). However, CSF is considered to be the best source of these biomarkers, because it is in direct contact with the brain and can better reflect the pathophysiological changes occurring in the central nervous system (CNS) (Olsson et al., 2016). Therefore, after controlling for age, gender, height, weight, BMI, years of education, history of smoking, history of drinking, hypertension, diabetes, coronary heart disease, ASA class, and other confounding factors, this study included patients undergoing total hip replacement under elective combined spinal and epidural anesthesia; preoperative CSF was measured by biochemical index assessment, and the concentration of related biomarkers was detected to further verify their important diagnostic value for POD.

SCD, also known as subjective memory impairment (SMI) and subjective cognitive impairment (SCI), was first established on the basis of AD research and was considered as the preclinical stage of AD (Jessen et al., 2014). Epidemiological studies have shown that the prevalence of SCD ranges from 10 to 88% and increases with age: 20% in people 65 years and younger, 25–50% in people 65 years and older, and 88% in people over 85 years of age (Gifford et al., 2015). For SCD, memory decline is the most common symptom, and at the same time it can be accompanied by visual–spatial impairment, language impairment, attention deficit, and other symptoms (Si et al., 2020). According to statistics, an estimated 27% of SCD patients will develop MCI in the future, and another 14% will eventually be diagnosed with cognitive impairment (Mitchell et al., 2014). At present, with the deepening of people's understanding of neuropathology, neuropsychology, neuroimaging, and pathophysiology, the study of SCD is no longer limited to AD but extends to the field of perioperative neurocognitive disorder (PND).

The results of this study showed that there were significant differences in the expression of Aβ40, Aβ42, P-tau, and T-tau in CSF between SCD Group and NC Group (*P* < 0.05), indicating an increased risk of POD in patients with SCD. Further logistic regression analysis of biomarkers in CSF showed that the increased concentrations of P-tau were a risk factor for POD, consistent with previous findings (Jia et al., 2019). At the same time, the concentrations of P-tau decreased, which indicated that the changes of biomarkers such as P-tau in CSF were also risk factors for SCD. There was an evidence that an increased tau protein in CSF deposition in SCD patients results in a decrease in glucose metabolism in the brain (Perrotin et al., 2012; Snitz et al., 2015; Buckley et al., 2017), a decrease in medial temporal lobe volume (Scheef et al., 2012), and a thinner cortex (Jessen et al., 2006; Saykin et al., 2006). In addition, the deposition of Tau proteins may be associated with the neurodegeneration that SCD patients are experiencing, and they interact to reduce CNS tolerance and ultimately accelerate the destruction of cognitive function (Hu et al., 2019). Combined with the results of this study, we hypothesized that the changes in the level of related biomarkers in patients with CSF might be one of the mechanisms of POD in patients with SCD.

A large number of studies have confirmed that advanced age is the main risk factor for SCD and POD. The reasons may be as follows: (1) in the elderly, brain atrophy, decreased number of neurons, degeneration of the central cholinergic system, and relative reduction of specific receptors and neurotransmitters lead to decreased learning, memory, and cognitive reserve. (2) The function of abnormal microglia or being in a “pre-excitation” state is more likely to be stimulated by surgery, anesthesia, and inflammation of peripheral tissues, leading to central inflammatory response. (3) Low organ function reserve, decreased vitality and increased vulnerability, and poor tolerance to anesthesia and surgery are prone to adverse drug reactions. (4) The permeability of the endothelium and blood–brain ridge fluid barrier is increased, and peripheral inflammatory factors enter the CNS to activate the central inflammatory response (Lucin et al., 2013; Rundshagen, 2014). In this study, patients will be included for stratified analysis according to their age. The study found that the incidence rate of POD in the SCD group increased with age, which was also confirmed to be an independent risk factor for POD in patients with SCD.

The limitations of this study are as follows: First, the number of patients was limited and more eligible patients would be included in future studies. Second, this study was a single-center study, which could be further validated by multicenter studies in the future. Third, this study only focused on the relationship between SCD and the biomarkers in patients' CSF and did not involve other related pathogenesis of POD, which depended on further research.

In conclusion, SCD is one of the preoperative risk factors for POD, which provides new insight into the prevention of POD. The early prevention of POD could improve the quality of patients' life and reduce the length of hospital stay and the burden on families and society.

## Data Availability Statement

The raw data supporting the conclusions of this article will be made available by the authors, without undue reservation.

## Ethics Statement

Ethical approval for this study (Ethical Committee N°2020 PRO FORMA Y number 005) was provided by the Ethical Committee Qingdao Municipal Hospital affiliated to Qingdao University, Qingdao, China (Chairman Prof Yang) on 21 May 2020. The patients/participants provided their written informed consent to participate in this study. Written informed consent was obtained from the individual(s) for the publication of any potentially identifiable images or data included in this article.

## Author Contributions

XL contributed to the study design, data collection, statistical analysis, and manuscript preparation. FL performed ELISA. BW was involved in the data collection. RD performed the neuropsychological testing. YB and MW contributed to the study concept and design, as well as manuscript preparation and review. XL and LS performed the statistical analysis. All authors contributed to the article and approved the submitted version.

## Conflict of Interest

The authors declare that the research was conducted in the absence of any commercial or financial relationships that could be construed as a potential conflict of interest.
